# The role of social support in reducing the impact of violence on adolescents’ mental health in São Paulo, Brazil

**DOI:** 10.1371/journal.pone.0258036

**Published:** 2021-10-06

**Authors:** Meaghen Quinlan-Davidson, Ligia Kiss, Delan Devakumar, Mario Cortina-Borja, Manuel Eisner, Maria Fernanda Tourinho Peres

**Affiliations:** 1 Institute for Global Health, University College London, London, United Kingdom; 2 Population, Policy and Practice Research and Teaching Department, Great Ormond Street Institute of Child Health, University College London, London, United Kingdom; 3 Institute of Criminology, University of Cambridge, Cambridge, United Kingdom; 4 Preventive Medicine Department, University of São Paulo, São Paulo, Brazil; University of Pennsylvania, UNITED STATES

## Abstract

**Objectives:**

We investigated whether perceived social support among adolescent students moderated the association between violence exposure and internalising symptoms in São Paulo city, Brazil.

**Methods:**

We tested the stress-buffering model using data from the cross-sectional school-based, survey São Paulo Project on the Social Development of Children and Adolescents. Internalising symptoms were measured using an adapted version of the Social Behaviour Questionnaire; serious victimisation, being bullied once/week, school violence and community violence, friend and teacher support were scales adapted by the research team; the Alabama Parenting Questionnaire measured parenting style. Linear mixed-effects models were used to quantify moderation effects of (i) social support between violence exposure and internalising symptoms and (ii) gender between violence exposure and internalising symptoms across schools.

**Results:**

Across schools, being bullied once/week, school violence, and community violence were associated with a significant (*p<*0.001) increase in internalising symptoms (e.g., bullied *b* = 5.76, 95% CI 2.26, 9.26; school violence *b* = 0.48, 95% CI 0.30, 0.67; community violence *b* = 0.36; 95% CI 0.22, 0.50). Males exposed to all types of violence had significantly lower (*p<*0.01) internalising symptoms compared to females (e.g., serious victimisation: *b* = -1.45; 95% CI -2.60, -0.29; school violence *b* = -0.27; 95% CI -0.30, -0.24; community violence *b* = -0.23; 95% CI -0.25, -0.20). As a main effect, social support was associated with a significant (*p<*0.01) decrease in internalising symptoms across schools (e.g., positive parenting *b* = -2.42; 95% CI -3.12, -1.72; parent involvement *b* = -2.75; 95% CI -3.32, -2.17; friend support *b* = -1.05; 95% CI -1.74, -0.34; teacher support *b* = -0.90; 95% CI -1.58, -0.22). Social support did not moderate the association between violence exposure and internalising symptoms.

**Conclusions:**

Adolescent students in São Paulo exposed to violence have a higher likelihood of internalising symptoms, compared to those who are not. Support from parents, friends, and teachers, independent of violence, appear to be protective against internalising symptoms, pointing to potential programmes that could improve adolescent mental health.

## Introduction

Exposure to violence in high-income countries is consistently associated with mental health problems [1], accumulating across the life-course [2] and especially during adolescence (10–19 years). This places adolescents at risk for poor mental health outcomes, such as anxiety, depression, interrupted sleep, and post-traumatic stress disorder (PTSD). Research suggests that internalising disorders, including depression and anxiety, are amongst the most common mental health outcomes of violence exposure among adolescents [3].

There are several types of violence that adolescents can experience, including bullying, assault by strangers, and violence related to property crimes. The World Health Organization (WHO) defines these types of violence as community violence, which includes all interpersonal violence not perpetrated by family members or intimate partners but by acquaintances and strangers [4]. However, little is known about the relationship between types of community violence and adolescent mental health in low and middle-income countries in general, and in Brazil specifically.

In Brazil, the national homicide rate is nearly five times the world average (30.5 vs 6.4 per 100,000 inhabitants) [5]. Adolescents are particularly at risk of violence as 51.8% of deaths among 15-19-year olds are due to homicides [6]. National estimates suggest that 19.2% of adolescents experience mental disorders [7]. In São Paulo city, the most populous city in the Americas, 7.5% of 12–19 year olds in a 2015 survey reported experiencing some type of violence (insult, threat, physical aggression) within the previous 12 months [8]. That same year, the prevalence of common mental disorders among adolescents (15–19 years) was estimated at 13.2% in the city [9]. Recent studies in São Paulo show that exposure to violence during childhood and adolescence are significantly associated with mental health problems, especially among individuals in low socioeconomic strata [10]. In addition, previous global research has demonstrated that adolescent boys have higher community violence exposure than girls [11], yet girls experience greater anxiety and depression when exposed to community violence than boys [12].

Social support and a supportive social environment, from community, family, and intimate relationships, play critical roles in promoting adolescents’ development and well-being [13]. It has been found to enhance physical or mental health while potentially reducing the effect of poor outcomes [13]. High levels of social support likely translate into positive outcomes for adolescents who are victims of violence through the development of new skills; by disclosing their experiences to parents, friends, and teachers, to help them cope [4]; and motivating adaptive behaviours [14]. Social support provides informational, emotional, and/or tangible resources, which can promote adaptive behavioural responses to acute or chronic stressors. A framework that conceptualises this relationship is the stress-buffering model. Buffering occurs in two ways: (i) if an individual perceives that positive support resources are available during a crisis, this improves their capability of handling the stressful situation; or (ii) the support available (from parents, friends, teachers) may provide a solution or lessen the perceived importance of the stressor, thereby reducing the negative reaction to the stressor on the individual’s health and behaviour [13].

Research from high-income countries suggests that social support could modify, or buffer, the relationship between violence exposure and poor mental health outcomes among adolescents [14, 15] although evidence is mixed. Evidence on adolescents in the United States (US) found that higher levels of social support moderated the association between violence exposure resulting in fewer internalising symptoms [16, 17]. In contrast, a number of studies from the US and Australia found no evidence of a moderating role of social support between violence and mental health [3, 18]. At the same time, violence may have an endogenous dynamic effect in schools and neighbourhoods [19]. For example, adolescents that attend a school where violence is pervasive, or live in a violent community, may feel the need to project an “aggressive persona” to protect themselves and maintain a sense of self-respect and identity [20]. Also, adolescents that perceive high levels of social support in school and their environment are more resilient to stressors and better able to adjust to challenges [21].

While substantial research has focussed on the association between violence exposure and internalising disorders among adolescents, evidence on factors that could protect adolescents from violence and poor mental health outcomes remains limited, particularly in low-and-middle-income countries (LMICs). In the current study, we first aim to explore whether gender moderates the association between violence exposure and internalising symptoms. We then aim to identify whether perceived social support (from parents, friends, and teachers) moderates the association between violence exposure (serious victimisation, being bullied once/week, school violence, and community violence) and adolescent internalising symptoms across schools in São Paulo city, Brazil. In doing so, the study tests the applicability of the stress-buffering model to adolescent students exposed to community violence.

Determining whether social support and gender are potential moderators of violence and internalising symptoms, and whether this relationship induces variability between schools, could have important public health and clinical implications to inform interventions and policies aimed at preventing or reducing the risk of internalising symptoms among adolescents exposed to violence [22]. In Brazil, where violence is frequent and pervasive, too few strategies and interventions exist to avoid and prevent adolescents’ exposure to violence and the resultant consequences.

## Materials and methods

**Fig 1:**
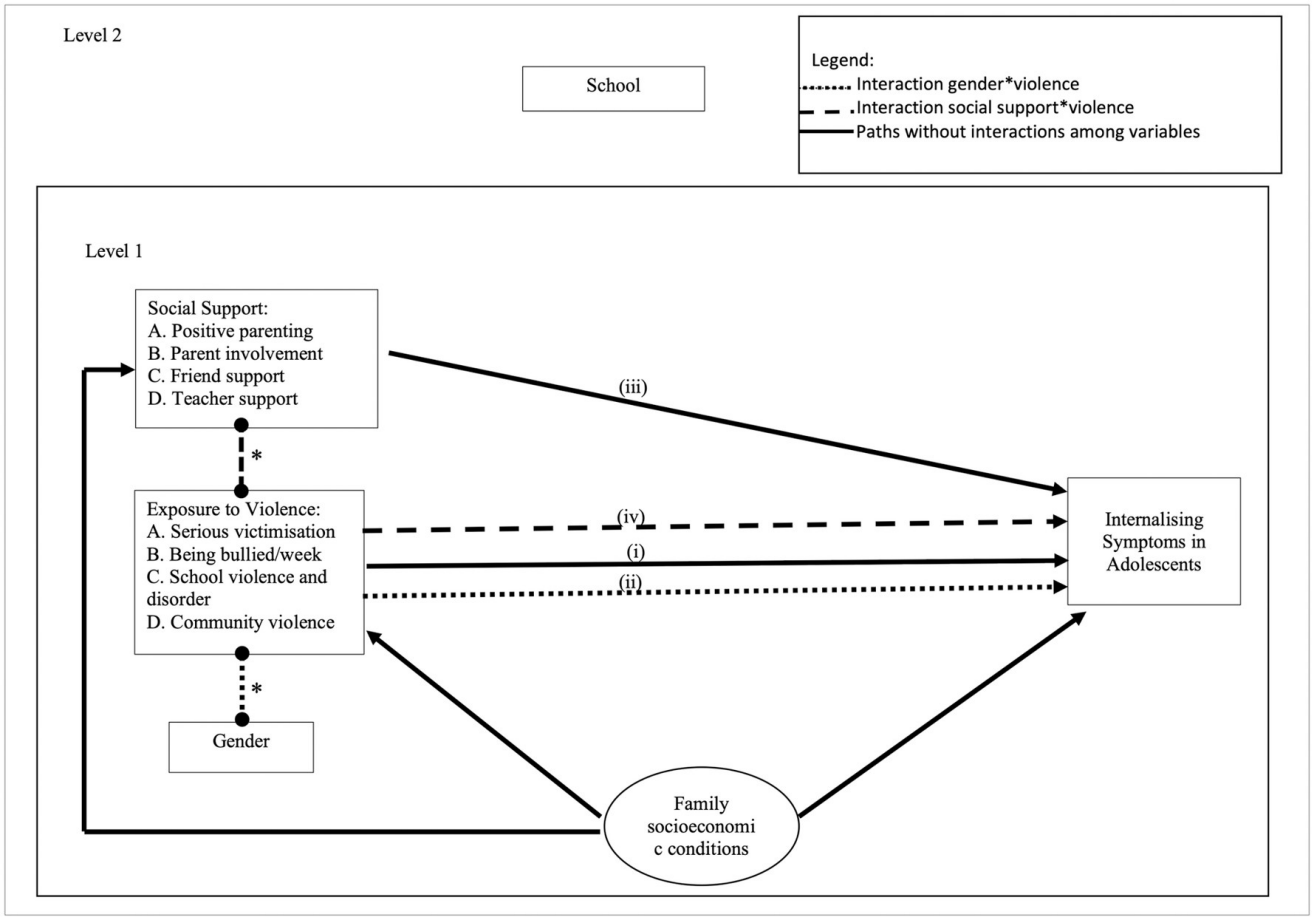
Model of exposure to violence, social support, internalising symptoms, and covariates.

We constructed a model, suggesting the direct effect of exposure to community violence, moderated by gender and social support, on internalising symptoms, adjusting for family socioeconomic status (SES) (Fig 1).

We conducted a secondary data analysis of the São Paulo Project on the Social Development of Children and Adolescents (SP-PROSO), a cross-sectional school-based study in São Paulo city, Brazil, conducted in 2017 [23]. A representative sample of adolescents attending their 9^th^ year of education were randomly selected, using stratified sampling with three strata of schools: public schools funded by the State of São Paulo, public schools funded by the city of São Paulo and private schools. The number of classes in each stratum was determined based on the number of adolescents enrolled by class and school in their 9^th^ year from the 2015 school census [24]. 156 classes from different schools were selected. Of these, 128 classes were selected for initial data collection. The remaining classes were to be used as a reserve to fulfil sample size requirements if the desired sample size was not reached. All of the schools selected for reserve were included due to a higher number of student absences on the day of data collection.

A total of 119 schools agreed to participate in the survey. Eligible adolescents were those present in the classroom on the day of data collection, whose parents did not proscribe their participation, and who did not appear seriously impaired in understanding the questions or answering anonymously, as assessed by teachers. The study sample consisted of 2,816 students; of which, 96 refused to participate and 18 were excluded due to failure to complete the questionnaire. This yielded a dataset of 2,702 adolescents aged 12.9 to 18.9 years.

The questionnaire was based on the instrument used in the sixth wave of the longitudinal Zurich Project on Social Development of Children [25] and the Montevideo Project for the Social Development of Children and Adolescents [26]. Details on the questionnaire have been published elsewhere [23]. Instrument translation and pre-testing were performed to maintain comparability and ensure the psychometric characteristics of the scales were not compromised [27]. The instruments were piloted among 116 students from public and private schools. We conducted reliability and confirmatory factor analysis (CFA) for all of the scales and found good fit for unidimensional solutions, as expected.

### Procedure

A printed copy of the questionnaire was given to adolescents for self-completion during classroom time. Trained researchers were present in the class for assistance and support. The questionnaires were anonymous for students and schools, with single identifiers of sequential numbers for each school and student. The questionnaires were reviewed upon completion to detect inconsistencies and missing values. If problems were detected, the researchers asked the student to complete the section again. A total of 2,680 adolescents answered more than 80% of the questionnaire (94.1% of the sample) and were included in the data analysis.

The study was approved by the University of São Paulo’s Committee for Ethics in Research and Brazil’s National Research Ethics Committee. Participation was voluntary and adolescents were provided with informed consent. A detailed description of the study’s protocol has been published elsewhere [23].

### Measures

#### Level 1-variables

*Internalising symptoms* were measured using an adapted 9-item sub-scale from the Social Behaviour Questionnaire (SBQ) [28], the internalising problem behaviour subscale. The scale measures mainly depressive and anxiety symptoms within the month prior to the survey, with one item on self-harm (internal consistency of internalising symptoms was Cronbach’s α: 0.84). Previous studies in Switzerland have evaluated the validity of the SBQ and results support the reliability, criterion validity, factorial validity and developmental invariance of the SBQ [29]. Participants responded using a five-point likelihood scale from “never” to “very often”. Answers to the questions were considered together, composing a summed, single measure. We analysed internalising symptoms as a continuous variable, as this captured the full range of internalising symptom levels while potentially detecting associations with variables in the models [29].

*Social support* was measured using:

1. A 14-item instrument adapted from the Alabama Parenting Questionnaire [30] was used to measure the following dimensions of parent support: positive parent style (internal consistency was Cronbach’s α: 0.66) and parent involvement (Cronbach’s α: 0.74). Positive parenting included 3-items. Parent involvement included 4-items. Participants answered each question using a 4-point likelihood scale from “never” to “often/always”. Two scores were created for positive parenting and parent involvement by averaging the items.
2. Friend support was measured using three questions developed by the Zurich project team, assessing the adolescent’s friendships within the previous year of the survey. Participants answered the questions on a 4-point likert scale from “totally disagree” to “totally agree”. A score for friend support was created by averaging the items [31].
3. Relationship to the teacher was measured using 3-items adapted by the Zurich project team with some items from the German Kriminologisches Forschungsinstitut Niedersachsen comparative studies on youth violence. Respondents answered the questions using a 4-point likert scale from “totally disagree” to “totally agree”. A score for teacher support was created by averaging the items [31].

*Violence Exposures* were measured using:

1. The Bullying Victim Index is a 5-item scale initially developed by Olweus [32] and adapted by the project team using Françoise Alsaker’s [33] definition of bully victimisation. The instrument measures being purposely ignored or excluded; laughed at, mocked or insulted. The items were selected to cover the range of bullying and are considered important forms of victimisation. The reliability and factorial validity of the index was evaluated in a previous study [34]. Although Solberg and Olweus [32] recommend a lower cut-off point of “2 or 3 times a month”, we included a stringent lower bound cut-off point of “once per week”. Respondents answered the questions using a 6-point likelihood scale from “never” to “almost every day”. A binary score of the maximum value of each item was computed of “yes, at least once per week” and “no”.
2. Serious victimisation was composed of 4-items, adapted by the project team on the basis of the Kriminologisches Forschungsinstitut Niedersachsen study [35]. The questions measure the prevalence of serious victimisation of adolescents within the previous 12-months of the survey. This included, victimisation of violence by robbery, assault with a weapon or object that led to injury. We created a binary measure of serious victimisation (no victimisation/victimisation).
3. Exposure to school violence and disorder was composed of 12-items and was created by the São Paulo project team. The questions measured the prevalence of witnessing or hearing about school violence, or school disorder, within the previous 12-months of the survey. Respondents answered the questions using a 4-point likelihood scale from “never” to “often (5+ times)”. A measure was computed as the sum of each item to make an aggregate result.
4. Exposure to community violence was composed of 14-items, adapted from “Children’s Exposure to Community Violence” [36]. The original scale had 10 items, however four items were added to explore types of violence common to the Brazilian reality, including witnessing or hearing about someone being murdered; carrying a weapon; someone that was bribed by police within the 12 months of the survey. Respondents answered the questions using a 4-point likelihood scale from “never” to “often (5+ times)”. A measure was computed as the sum of each item to make an aggregate result.

*Covariates*. We included gender (male/female) and family socioeconomic conditions (low, medium, high). Relative family socioeconomic conditions were measured using the socioeconomic score from the Brazilian Institute of Geography and Statistics, used in the Brazilian National Surveys of School Health [37]. This score is composed of items on maternal educational level, family assets (e.g. TV, computer, vehicle) and access to services (e.g. telephone, paid domestic workers). Each item was weighted by the inverse of the prevalence in the sample. A score was computed from the from the sum of those weights for each adolescent, and the resulting variable was divided in tertiles [38].

#### Level 2-variable

Schools were identified by a number between 1 and 119.

### Statistical methods

As the primary sampling unit for the study was the school, we weighted the sample at both levels, adjusting for the clustered survey design and accounting for the varying number of students per school [23]. Cronbach’s α was used to assess internal consistency of the scales, and goodness-of-fit was assessed for factor analysis models using factor extraction and uniqueness values. Factorial and construct validity were measured using single-factor CFA models for all scales. Descriptive summaries, including Pearson’s correlation coefficients were calculated for all study variables. We also calculated Pearson’s correlation coefficients for the social support scores. Following Rabe-Hesketh and colleagues (2012) [39], we tested the random effects variance, or whether each type of violence exposure score, social support score and internalising score varied between schools, using a likelihood-ratio (LR) test. We also tested whether multilevel modelling was needed by assessing the LR of the nested and final models. To assess the variance between schools, we estimated the intraclass correlation coefficient (ICC) for the model with internalising symptoms only and final models. We used Wald’s chi-squared tests with a Bonferroni-type adjustment following the approach from Korn and Graubard [40] to determine the significance of each model’s variance components. Note that these adjustments refer only to the significance tests of the variance components in the models, not to possible multiple comparisons which could be derived by the contrasts in the models’ fixed effects terms. To assess the degree to which each type of social support score differs from each other, we analysed the discriminant validity by investigating the correlations between items and scores or rest-scores as recommended by Perrot and colleagues (2018) [41].

To test the stress-buffering model, we fitted two-level linear mixed-effects (LME) models across schools. We assessed on the first level: (i) the effect of each type of violence exposure on internalising symptoms; (ii) the interactive effect of each type of violence exposure and gender on internalising symptoms (i.e., × gender(male)); (iii) the effect of perceived social support on internalising symptoms; and (iv) the interactive effect between each type of violence exposure and perceived social support on internalising symptoms (e.g., × positive parenting). We assessed variation within- and between- schools (second level), using maximum likelihood estimation and robust standard errors [39]. Random effects terms were included in the models’ intercepts. We conducted post-hoc simple effect and simple contrasts analyses to determine the difference in internalising symptoms between girls and boys exposed to violence. Model selection was performed using the Bayesian Information Criterion (BIC). Smaller BICs indicated increased model fit penalised by the model’s complexity. We also analysed all of the four sources of social support together in each LME model to determine if there was a difference in their association with internalising symptoms. Family SES was controlled for in all models. Stata version 15.1 was used [42].

## Results

Study participants’ characteristics and the adjusted proportions for internalising symptoms, violence, and social support among all participants are presented in Table 1.

**Table 1:** Characteristics of study participants and mean of internalising symptoms, violence, and social support among study participants (weighted).

	Distribution		Mean(SD)	
	*n*	%	All	Range
Gender (*n* = 2,551)				
Male	1,323	52.6		
Female	1,228	47.4		
Race^a^ (*n* = 2,597)				
White	1,163	44.2		
Black, Brown	1,268	49.0		
Yellow	100	4.4		
Indigenous	66	2.3		
Internalising Symptoms (*n* = 2,611)			23.42 (7.87)	8–45
Depression			11.10 (4.07)	1–20
Anxiety			10.84 (3.95)	4–20
Self-harm			1.49 (1.05)	1–5
Violence				
Serious victimisation (*n* = 2,614)			0.23 (0.42)	0–1
Bullied at least once per week (*n* = 2,607)			0.23 (0.42)	0–1
School violence (*n* = 2,617)			21.29 (6.30)	2–48
Community violence (*n* = 2,617)			24.17 (8.93)	4–56
Perceived Social Support				
Positive parenting (*n* = 2,615)			2.98 (0.66)	1–4
Parent involvement (*n* = 2,615)			2.90 (0.76)	1–4
Friend support (*n* = 2,614)			3.28 (0.61)	1–4
Teacher support (*n* = 2,600)			2.89 (0.60)	1–4

^a^Race in Brazil is measured as phenotypic skin colour and ethnicity as explained in Travassos C & Williams DR. The concept and measurement of race and their relationship to public health: a review focused on Brazil and the United States. Cad. Saude Publica, Rio de Janeiro. 2004; 20(3): 660–678.

Cronbach’s α for the following indices were: Bully-Victim (α = 0.70), serious victimisation (α = 0.51), school violence (α = 0.83), community violence (α = 0.82), positive parenting (α = 0.66), and parent involvement (α = 0.74). Pearson’s correlation coefficients showed that internalising symptoms were significantly associated with increased serious violence victimisation within the previous year (*r* = 0.17, *p<*0.001), being bullied once/week (*r* = 0.28, *p<*0.001), school violence (*r* = 0.29, *p<*0.001) and community violence (*r* = 0.21, *p<*0.001) within the year prior. Internalising symptoms were also significantly associated with decreased levels of perceived positive parenting (*r* = -0.22, *p<*0.001), parent involvement (*r* = -0.27, *p<*0.001), friend support (*r* = -0.09, *p<*0.001), and being male (*r* = -0.38, *p<*0.001). Pearson’s correlation coefficients showed that the social support scales were all significantly correlated with each other (*p*<0.001); positive parenting was correlated with parent involvement (*r* = 0.65), while the correlation coefficients for positive parenting, parent involvement, friend support and teacher support scales were smaller (*r* ≤ 0.22) (S1 Table). The ICC for the model including only internalising symptoms was 0.73% (95% CI 0.1%, 4.2%), suggesting that internalising symptoms do not vary much across schools. Table 2 illustrates the ICC results of the model with internalising symptoms only and final models.

**Table 2:** Intraclass correlation coefficient of model with internalising symptoms only and final models at the school level (weighted).

	ICC	SE	95% CI	
Model including only internalising symptoms	0.007	0.007	0.001	0.04
Serious Victimisation × Positive Parenting	0.009	0.008	0.002	0.05
Serious Victimisation × Parent Involvement	0.010	0.007	0.002	0.04
Serious Victimisation × Friend Support	0.010	0.008	0.002	0.04
Serious Victimisation × Teacher Support	0.008	0.007	0.002	0.04
Bullied × Positive Parenting	0.008	0.008	0.001	0.05
Bullied × Parent Involvement	0.008	0.008	0.001	0.05
Bullied × Friend Support	0.008	0.007	0.001	0.04
Bullied × Teacher Support	0.007	0.007	0.001	0.05
School Violence × Positive Parenting	0.010	0.008	0.004	0.04
School Violence × Parent Involvement	0.010	0.009	0.004	0.05
School Violence × Friend Support	0.020	0.008	0.005	0.04
School Violence × Teacher Support	0.010	0.008	0.004	0.04
Neighbourhood Violence × Positive Parenting	0.010	0.009	0.003	0.05
Neighbourhood Violence × Parent Involvement	0.010	0.009	0.003	0.05
Neighbourhood Violence × Friend Support	0.010	0.008	0.003	0.04
Neighbourhood Violence × Teacher Support	0.010	0.009	0.003	0.05

Following Korn and Graubard’s approach [40], we found that internalising symptoms, violence, social support and gender did not vary significantly between schools (Tables 3–6). There were three schools in low income neighbourhoods in the East and South of São Paulo city that had significantly higher levels (*p* < 0.01) of internalising symptoms (Vila Carmosina, m: 28.05, SD: 9.41; Parque Ligia, m: 28.68, SD: 11.76; and Artur Alvim, m: 27.13, SD: 9.13). In contrast, one school in a wealthier, central neighbourhood of the city and another in a middle-class neighbourhood in the south of the city reported significantly lower levels (*p*≤0.02) of internalising symptoms, respectively (Vila Uberabinha, m: 19.83, SD: 5.44 and Campininha, m: 19.39, SD: 4.51).

**Table 3:** Linear mixed-effects models of serious victimisation, type of social support & interactions (weighted).

	Internalising Symptoms				
	Coefficient	SE	95% CI		*p*-value
**Model 1. Positive Parenting**					
Serious Victimisation	3.03	1.93	-0.75	6.80	0.120
Positive Parenting	-2.42	0.36	-3.12	-1.72	<0.001
Male	-5.64	0.39	-6.39	-4.89	<0.001
Serious victimisation × Positive Parenting	0.20	0.64	-1.06	1.46	0.760
Serious victimisation × Gender (Male)	-1.51	0.63	-2.75	-0.27	0.020
Contrast (Male/Female) exposed	-7.15	0.58	-8.28	-6.02	<0.001
Variance (School)	0.45	0.38	0.09	2.37	1.00*
Variance (Student)	48.16	1.64	45.06	51.48	
**Model 2. Parent Involvement**					
Serious Victimisation	2.73	1.59	-0.39	5.85	0.090
Parent Involvement	-2.75	0.29	-3.32	-2.17	<0.001
Male	-5.90	0.39	-6.66	-5.14	<0.001
Serious victimisation × Parent Involvement	0.24	0.52	-0.78	1.25	0.650
Serious victimisation × Gender (Male)	-1.45	0.59	-2.60	-0.29	0.010
Contrast (Male/Female) exposed	-7.35	0.53	-8.38	-6.31	<0.001
Variance (School)	0.46	0.34	0.11	1.95	0.88*
Variance (Student)	46.52	1.57	43.53	49.71	
**Model 3. Friend Support**					
Serious Victimisation	3.66	2.55	-1.34	8.66	0.150
Friend Support	-1.05	0.36	-1.74	-0.34	<0.001
Male	-5.66	0.40	-6.44	-4.88	<0.001
Serious victimisation × Friend Support	0.14	0.72	-1.28	1.55	0.850
Serious victimisation × Gender (Male)	-1.77	0.65	-3.05	-0.50	0.01
Contrast (Male/Female) exposed	-7.43	0.59	-8.58	-6.28	<0.001
Variance (School)	0.52	0.38	0.13	2.17	1.00*
Variance (Student)	49.90	1.72	46.64	53.39	
**Model 4. Teacher Support**					
Serious Victimisation	3.76	2.17	-0.49	8.01	0.080
Teacher Support	-0.90	0.35	-1.58	-0.22	0.010
Male	-5.75	0.41	-6.54	-4.95	0.010
Serious victimisation × Teacher Support	0.13	0.68	-1.20	1.45	0.850
Serious victimisation × Gender (Male)	-1.87	0.67	-3.18	-0.56	0.010
Contrast (Male/Female) exposed	-7.62	0.62	-8.83	-6.41	<0.001
Variance (School)	0.42	0.36	0.08	2.29	0.96*
Variance (Student)	50.18	1.71	46.92	53.65	

*Wald chi-squared statistic using a Bonferroni-type adjustment testing the model’s variance components.

**Table 4:** Linear mixed-effects models of bullied once per week, type of social support & interactions (weighted).

	Internalising Symptoms				
	Coefficient	SE	95% CI		*p*-value
**Model 1. Positive Parenting**					
Bullied	5.76	1.78	2.26	9.26	0.001
Positive Parenting	-2.24	0.31	-2.85	-1.63	<0.001
Male	-5.38	0.35	-6.07	-4.68	0.030
Bullied × Positive Parenting	-0.12	0.63	-1.36	1.12	0.850
Bullied × Gender (Male)	-1.74	0.80	-3.31	-0.17	0.030
Contrast (Male/Female) bullied	-7.12	0.77	-8.62	-5.62	<0.001
Variance (School)	0.35	0.35	0.05	2.48	0.88*
Variance (Student)	46.03	1.57	43.06	49.21	
**Model 2. Parent Involvement**					
Bullied	5.34	1.44	2.51	8.17	<0.001
Parent Involvement	-2.53	0.27	-3.06	-1.99	<0.001
Male	-5.73	0.36	-6.44	-5.02	<0.001
Bullied × Parent Involvement	-0.13	0.54	-1.19	0.93	0.81
Bullied × Gender (Male)	-1.21	0.81	-2.79	0.38	0.14
Contrast (Male/Female) bullied	-6.94	0.76	-8.42	-5.45	<0.001
Variance (School)	0.38	0.35	0.06	2.35	0.89*
Variance (Student)	44.56	1.49	41.73	47.57	
**Model 3. Friend Support**					
Bullied	5.95	1.81	2.40	9.50	0.001
Friend Support	-0.67	0.35	-1.36	0.03	0.060
Male	-5.47	0.37	-6.19	-4.74	<0.001
Bullied × Friend Support	-0.11	0.57	-1.22	1.00	0.850
Bullied × Gender (Male)	-1.61	0.80	-3.19	-0.04	0.040
Contrast (Male/Female) bullied	-7.08	0.78	-8.61	-5.56	<0.001
Variance (School)	0.36	0.33	0.06	2.16	0.80*
Variance (Student)	47.92	1.62	44.85	51.21	
**Model 4. Teacher Support**					
Bullied	6.61	1.62	3.43	9.79	<0.001
Teacher Support	-0.69	0.32	-1.33	-0.06	0.030
Male	-5.55	0.38	-6.29	-4.82	<0.001
Bullied × Teacher Support	-0.30	0.56	-1.39	0.80	0.590
Bullied × Gender (Male)	-1.69	0.80	-3.25	-0.13	0.030
Contrast (Male/Female) bullied	-7.24	0.77	-8.75	-5.73	<0.001
Variance (School)	0.32	0.33	0.04	2.38	0.79*
Variance (Student)	47.86	1.58	44.86	51.07	

*Wald chi-squared statistic using a Bonferroni-type adjustment testing the model’s variance components.

**Table 5:** Linear mixed-effects models of school violence, type of social support & interactions (weighted).

	Internalising Symptoms				
	Coefficient	SE	95% CI		*p-*value
**Model 1. Positive Parenting**					
School Violence	0.48	0.10	0.30	0.67	<0.001
Positive Parenting	-2.09	0.72	-3.50	-0.68	0.004
Male	-1.35	1.16	-3.62	0.93	0.250
School violence × Positive Parenting	-0.01	0.03	-0.07	0.04	0.660
School violence × Gender (Male)	-0.26	0.01	-0.29	-0.23	<0.001
Contrast (Male/Female) exposed	-1.35	1.16	-3.62	0.93	0.250
Variance (School)	0.64	0.39	0.19	2.09	1.00*
Variance (Student)	45.57	1.56	42.60	48.74	
**Model 2. Parent Involvement**					
School Violence	0.38	0.12	0.16	0.61	0.001
Parent Involvement	-3.05	0.84	-4.70	-1.40	<0.001
Male	-1.92	1.10	-4.08	0.24	0.080
School violence × Parent Involvement	0.02	0.04	-0.05	0.09	0.580
School violence × Gender (Male)	-0.27	0.01	-0.30	-0.24	<0.001
Contrast (Male/Female) exposed	-1.92	1.10	-4.08	0.24	0.080
Variance (School)	0.68	0.39	0.23	2.08	1.00*
Variance (Student)	44.12	1.50	41.28	47.15	
**Model 3. Friend Support**					
School Violence	0.38	0.13	0.13	0.64	0.003
Friend Support	-1.75	0.83	-3.36	-0.09	0.040
Male	-1.52	1.12	-3.71	0.67	0.170
School violence × Friend Support	0.03	0.04	-0.05	0.10	0.480
School violence × Gender (Male)	-0.26	0.02	-0.29	-0.23	<0.001
Contrast (Male/Female) exposed	-1.52	1.12	-3.71	0.67	0.170
Variance (School)	0.78	0.40	0.29	2.11	1.00*
Variance (Student)	47.24	1.63	44.15	50.56	
**Model 4. Teacher Support**					
School Violence	0.32	0.11	0.11	0.53	0.002
Teacher Support	-1.82	0.84	-3.47	-0.17	0.030
Male	-1.64	1.12	-3.84	0.57	0.150
School violence × Teacher Support	0.05	0.04	-0.02	0.12	0.140
School violence × Gender (Male)	-0.26	0.02	-0.29	-0.23	<0.001
Contrast (Male/Female) exposed	-1.64	1.12	-3.84	0.57	0.140
Variance (School)	0.70	0.40	0.23	2.13	1.00*
Variance (Student)	47.6	1.61	44.56	50.89	

*Wald chi-squared statistic using a Bonferroni-type adjustment testing the model’s variance components.

**Table 6:** Linear mixed-effects models of exposure to community violence, type of social support & interactions (weighted).

	Internalising Symptoms				
	Coefficient	SE	95% CI		*p*-value
**Model 1. Positive Parenting**					
Community violence	0.36	0.07	0.22	0.50	<0.001
Positive Parenting	-1.77	0.62	-2.99	-0.54	0.005
Male	-3.99	1.00	-5.96	-2.03	<0.001
Community violence × Positive Parenting	-0.03	0.02	-0.07	0.02	0.230
Community violence × Gender (Male)	-0.22	0.01	-0.25	-0.19	<0.001
Contrast (Male/Female) exposed	-3.99	1.00	-5.96	-2.03	<0.001
Variance (School)	0.56	0.42	0.13	2.41	1.00*
Variance (Student)	47.65	1.54	44.73	50.76	
**Model 2. Parent Involvement**					
Community violence	0.28	0.06	0.16	0.41	<0.001
Parent Involvement	-2.60	0.59	-3.77	-1.44	<0.001
Male	-4.73	0.98	-6.65	-2.80	<0.001
Community violence × Parent Involvement	<-0.01	0.02	-0.04	0.04	0.900
Community violence × Gender (Male)	-0.23	0.01	-0.25	-0.20	<0.001
Contrast (Male/Female) exposed	-4.73	0.98	-6.65	-2.80	<0.001
Variance (School)	0.58	0.41	0.15	2.28	1.00*
Variance (Student)	46.23	1.48	43.42	49.23	
**Model 3. Friend Support**					
Community violence	0.21	0.09	0.04	0.38	0.010
Friend Support	-1.49	0.67	-2.80	-0.19	0.020
Male	-4.54	1.04	-6.57	-2.51	<0.001
Community violence × Friend Support	0.02	0.03	-0.03	0.08	0.360
Community violence × Gender (Male)	-0.22	0.01	-0.25	-0.19	<0.001
Contrast (Male/Female) exposed	-4.54	1.04	-6.57	-2.51	<0.001
Variance (School)	0.57	0.40	0.14	2.25	1.00*
Variance (Student)	49.85	1.60	46.81	53.08	
**Model 4. Teacher Support**					
Community violence	0.34	0.07	0.20	0.48	<0.001
Teacher Support	-0.22	0.73	-1.66	1.22	0.770
Male	-4.45	1.03	-6.46	-2.43	<0.001
Community violence × Teacher Support	-0.02	0.02	-0.06	0.03	0.500
Community violence × Gender (Male)	-0.22	0.01	-0.25	-0.19	<0.001
Contrast (Male/Female) exposed	-4.45	1.03	-6.46	-2.43	<0.001
Variance (School)	0.49	0.39	0.10	2.33	1.00*
Variance (Student)	50.03	1.60	46.99	53.27	

*Wald chi-squared statistic using a Bonferroni-type adjustment testing the model’s variance components.

The results of the discriminant validity test on the social support scores revealed that items of positive parenting and parent involvement exceeded a correlation threshold of r>0.4 [41], implying a good representation of parent support. All other items had a correlation coefficient with the score of their own dimension greater than those computed with other social support scores. These results indicate that positive parenting and parent involvement are separate constructs from friend support and teacher support (S2 Table).

Tables 3–6 show the LMEs estimates of the following fixed effects (level 1) on internalising symptoms: (i) the main effect of each exposure to violence; (ii) the main effect of each source of social support; (iii) the main effect of gender; (iv) the interactive effects of violence and source of social support; (v) the interactive effects of violence and gender; and (vi) the random effects across schools, expressed as variance between schools (level-2). Tables 7–10 show the LME estimates of the following fixed effects (level 1) on internalising symptoms: (i) the main effect of each exposure to violence; (ii) the main effect of all four sources of social support; (iii) the main effect of gender; and (vi) the random effects across schools, expressed as variance between schools (level-2).

**Table 7:** Linear mixed-effects models of serious victimisation & all sources of social support (weighted).

	Internalising Symptoms				
	Coefficient	SE	95% CI		*p*-value
Serious Victimisation	2.55	0.34	1.90	3.21	<0.001
Positive Parenting	-0.75	0.38	-1.50	0.00	0.050
Parent Involvement	-2.24	0.33	-2.88	-1.59	<0.001
Friend Support	-0.26	0.32	-0.89	0.37	0.420
Teacher Support	0.03	0.34	-0.65	0.70	0.940
Male	-6.18	0.34	-6.85	-5.51	<0.001
Variance (School)	0.46	0.36	0.10	2.11	0.95*
Variance (Student)	46.25	1.56	43.29	49.41	

*Wald chi-squared statistic using a Bonferroni-type adjustment testing the model’s variance components.

**Table 8:** Linear mixed-effects models of bullied once per week & all sources of social support (weighted).

	Internalising Symptoms				
	Coefficient	SE	95% CI		*p*-value
Bullied	4.34	0.39	3.57	5.10	<0.001
Positive Parenting	-0.71	0.37	-1.43	0.01	0.050
Parent Involvement	-2.19	0.32	-2.81	-1.57	<0.001
Friend Support	0.03	0.32	-0.61	0.66	0.940
Teacher Support	0.03	0.33	-0.62	0.68	0.930
Male	-5.97	0.34	-6.64	-5.31	<0.001
Variance (School)	0.38	0.36	0.06	2.46	0.93*
Variance (Student)	44.30	1.45	41.54	47.25	

*Wald chi-squared statistic using a Bonferroni-type adjustment testing the model’s variance components.

**Table 9:** Linear mixed-effects models of school violence & all sources of social support (weighted).

	Internalising Symptoms				
	Coefficient	SE	95% CI		*p*-value
School Violence	0.29	0.03	0.24	0.34	<0.001
Positive Parenting	-0.75	0.35	-1.44	-0.06	0.030
Parent Involvement	-2.16	0.31	-2.77	-1.54	<0.001
Friend Support	-0.42	0.30	-1.02	0.17	0.160
Teacher Support	0.19	0.33	-0.45	0.83	0.560
Male	-5.80	0.34	-6.47	-5.14	<0.001
Variance (School)	0.60	0.39	0.17	2.12	1.00*
Variance (Student)	44.14	1.55	41.21	47.29	

*Wald chi-squared statistic using a Bonferroni-type adjustment testing the model’s variance components.

**Table 10:** Linear mixed-effects models of exposure to community violence & all sources of social support (weighted).

	Internalising Symptoms				
	Coefficient	SE	95% CI		*p*-value
Neighbourhood Violence	0.15	0.02	0.12	0.18	<0.001
Positive Parenting	-0.70	0.38	-1.44	0.03	0.060
Parent Involvement	-2.30	0.32	-2.93	-1.67	<0.001
Friend Support	-0.23	0.32	-0.85	0.39	0.480
Teacher Support	0.15	0.33	-0.49	0.79	0.660
Male	-5.97	0.35	-6.65	-5.29	<0.001
Variance (School)	0.58	0.42	0.14	2.39	1.00*
Variance (Student)	45.41	1.48	42.60	48.39	

*Wald chi-squared statistic using a Bonferroni-type adjustment testing the model’s variance components.

In all adjusted models, being bullied once/week, school violence and community violence were significantly associated with an increase in internalising symptoms (Tables 4–6) while serious victimisation was not (Table 3). For example, according to Model 1 of Table 4, for each student that reported being bullied once per week, this was associated with a significant increase in internalising symptoms (*p*<0.001) of 5.76 points, controlling for all other covariates. For students that reported experiencing positive parenting, this was associated with a significant (*p*<0.001) decrease in internalising symptoms of 2.24 points, with all other covariates controlled for. For male students who reported being bullied once per week, this was associated with a significant (*p* = 0.03) decrease in internalising symptoms of 5.38 points, controlling for all other covariates.

Across all adjusted models with interactions, social support from all sources, independently of its interactive effect with exposure to violence, was significantly associated with a decrease in internalising symptoms (Tables 3–6). Across the adjusted models with all sources of social support included, parent involvement and positive parenting were significantly associated with a decrease in internalising symptoms in those exposed to serious victimisation, bullied once per week and exposed to school violence (*p*<0.05) (Tables 7–9). Parent involvement was significantly associated with a decrease in internalising symptoms in those exposed to community violence (*p*<0.001) (Table 10). Family SES was significant when students reported exposure to school violence.

**Fig 2:**
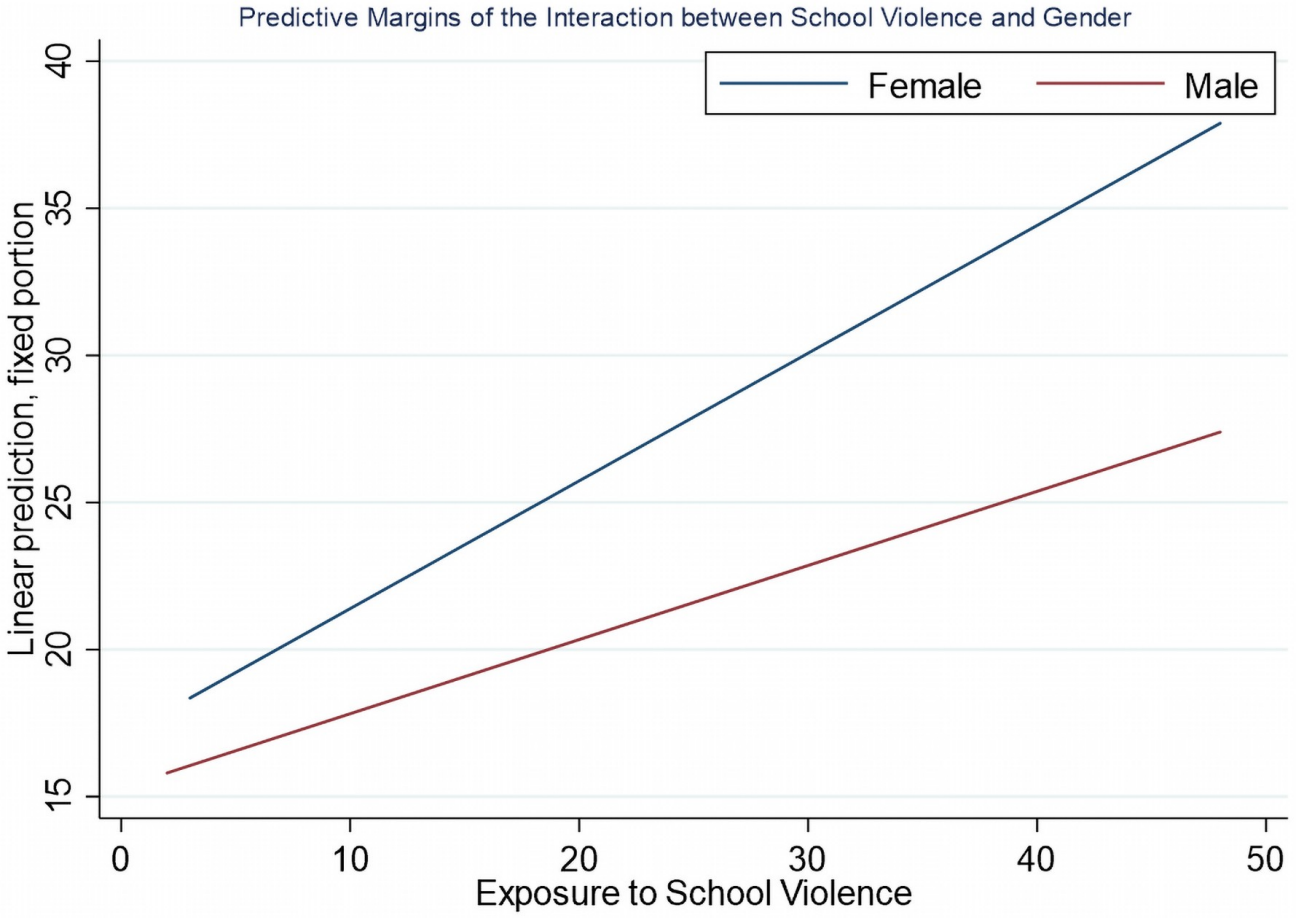
Gender as a moderator between exposure to school violence and internalising symptoms.

We found that gender significantly moderated the association between exposure to violence and internalising symptoms in most of the adjusted models. Boys who experienced serious victimisation (× positive parenting: *b* = -1.51, CI: -2.75- -0.27; × parent involvement: *b* = -1.45, CI: -2.60- -0.29; × friend support: *b* = -1.77, CI: -3.05- -0.50; × teacher support: *b* = -1.87, CI: -3.18- -0.56), were bullied once/week (× positive parenting: *b* = -1.74, CI: -3.31- -0.17; × friend support: *b* = -1.61, CI: -3.19- -0.04; × teacher support: *b* = -1.69, CI: -3.25- -0.13), school violence (× positive parenting: *b* = -0.20, CI: -0.30- -0.10; × parent involvement: *b* = -0.18, CI: -0.28- -0.09; × friend support: *b* = -0.19, CI: -0.29- -0.10; × teacher support: *b* = -0.19, CI: -0.29- -0.09) and community violence (× positive parenting: *b* = -0.07, CI: -0.15–0.0001) had significantly lower internalising symptoms compared to girls (Tables 2–4 and 7). Boy’s report of exposure to serious victimisation was not significantly different to girls (12.2% versus 10.6%, *p =* 0.48), while there was a significant difference in reported exposure to being bullied once/week (10.6% boys and 11.8% girls, *p =* 0.05), school violence (30.8% boys, 33% girls, *p<*0.001) and community violence (30.3% boys, 32% girls, *p<*0.001). Post-hoc simple effects contrast analyses found that boys exposed to serious victimisation, bullied once/week, and experienced community violence had significantly lower internalising symptoms compared to girls who had similar experiences (Tables 2–4). To show an example of what the interactions look like, Fig 2 illustrates how gender moderates the relationship between school violence on internalising symptoms.

We did not find that positive parenting, parent involvement, friend support, and teacher support moderated the effect of any type of exposure to violence and internalising symptoms among adolescent students.

The variance between and within schools was calculated. The variance of the random effects due to differences between schools in violence, social support and covariates ranged between 0.35 and 0.57. The variance of the random effects due to differences between students (after adjusting for exposure to violence, social support, and covariates) ranged from 44.49 to 50.34.

## Discussion

We investigated the potential role of perceived social support in protecting adolescents in São Paulo city, Brazil, from the negative effect of exposure to different types of violence on internalising symptoms. We examined different sources of social support to understand if and how each modified the association between violence exposure and internalising symptoms. We found that all types of violence exposure increased the risk of internalising symptoms, except for serious victimisation. We also found that gender significantly moderated the association between exposure to violence and internalising symptoms. And that different sources of social support significantly decreased the likelihood of internalising symptoms among adolescent students. However, we did not find that social support moderated the association between exposure to different types of violence and internalising symptoms.

Our findings did not support the Stress Buffering Model when applied to the role of social support in the relationship between violence and internalising symptoms. Prior research has reached mixed conclusions, with some US studies showing similar results that social support did not moderate the association between violence exposure and mental health issues [3, 16, 18]. Hammack and colleagues (2004) found that social support failed to protect against poor mental health outcomes when adolescents were exposed to violence [3]. Our findings are similar to the those initially put forth by Luthar (2000), who interpreted that perceived social support buffers against internalising symptoms when stress is low but not when it is elevated [43]. The role of social support in the relation between violence and internalising symptoms could also be related to the type and intensity of the violence victimisation. It has been posited that the emotional toll of being a victim of violence could suppress the positive effects of social support [3].

Prior literature has found that adolescent girls are more likely to experience internalising symptoms compared to boys when exposed to community violence [11]. Although boys exposed to violence were significantly less likely to have internalising symptoms, this could be attributed to gender differences in the way girls and boys experience, process, and express trauma. Contrary to prior literature [11, 44, 45], we found that girls reported greater exposure to being bullied once/week, school violence and community violence as compared to boys. This highlights a need for a thorough assessment of exposure to community violence by gender, and potentially greater intervention targeting of girls exposed to violence.

We found that social support was inversely related to internalising symptoms among students in all adjusted models. Positive parenting and parent involvement played a significant role in decreasing internalising symptoms. Prior literature from the US has found similar results, with high levels of parent participation, involvement, and overall support associated with decreased levels of depression, anxiety, and PTSD among adolescents [3, 16]. It has been posited that when adolescents are exposed to violence, they seek support from their parents, which helps the adolescent develop coping mechanisms, enables access and promotes supportive resources, and encourages competence in handling issues [46]. Friend support was also significantly associated with decreased internalising symptoms among students. Prior literature shows that support from friends can serve to promote exploration, the adolescent’s own self-worth, satisfaction of socio-affective needs (e.g., affection, love, attachment, loyalty, security), and new skill development [47]. Teacher support was found to decrease the likelihood of internalising symptoms. As school plays a large role in adolescents’ lives, teacher support could promote positive mental health outcomes within the school context [18]. These findings suggest that social support may lessen negative mental health effects; and policies and programs that focus on promoting and improving social support could be beneficial.

We also found that social support was inversely related to violence exposure. This is in line with previous evidence, which shows that social support can protect against negative experiences, such as violence [3, 48–52]. Social support can help the adolescent cope with violence victimisation through advice, boosting self-esteem and ensuring that the adolescent maintains a feeling of confidence [48]. It also helps adolescents disclose their experiences to their parents, friends and teachers, which could help them cope with stressful events [3].

We acknowledge some of the study limitations. Adolescents enrolled in school and present on the day of the survey were included; they may be different from those absent or not enrolled in school. The study did not investigate the quality of the support received, the perceived amount of support given, or the support provided. Due to the cross-sectional design of the study, we were unable to investigate reverse causality between poor mental health and violence exposure, and social support and violence exposure. We measured school and community violence through adolescents’ perceptions and not with objective indicators (i.e., homicide rates, the number of police apprehensions). We assessed perceived social support as general tendencies, instead of specific responses to the exposure experienced. We note that the serious victimisation scale has lower internal consistency (α = 0.51) than the other scales. This may have been driven by the first and second factor, however, we have included all items as removing any item from the scale would result in a smaller α. We acknowledge that this was an observational study and that the measurement level of the outcomes was ordinal, therefore, conclusions about absolute changes are subject to this constraint.

The study offers a unique contribution to the literature on adolescents living in São Paulo city and the associations between social support, violence, and internalising symptoms. It offers a starting point for future in-depth quantitative and qualitative studies on this topic in São Paulo city. Our analysis shows that social support may play a significant role in lowering the likelihood of developing internalising symptoms among adolescents, particularly females. Due to the pervasiveness of violence in Brazil, and that there are few strategies for adolescents to avoid exposure to violence, support from parents, friends, and teachers could potentially be interventions to prevent poor mental health outcomes among this age group.

## Supporting information

S1 Table Pearson’s correlation coefficients of social support variables (weighted). (DOCX)

S2 Table Discriminant validity of social support scales (weighted). (DOCX)
