# Supplementary material for: The role of social support in reducing the impact of violence on adolescents’ mental health in São Paulo, Brazil
Source: PLoS One. 2021 Oct 6;16(10):e0258036. doi: 10.1371/journal.pone.0258036 (PMC8494303; doi:10.1371/journal.pone.0258036)
Supplement: S1 Table — (DOCX) [file pone.0258036.s001.docx]

S1 Table. Pearson's Correlation Coefficients of Social Support Variables (weighted)

|  | Positive parenting | Parent involvement | Friend support | Teacher support |
| --- | --- | --- | --- | --- |
| Positive parenting | 1.00 |  |  |  |
| Parent involvement | 0.65*** | 1.00 |  |  |
| Friend support | 0.20*** | 0.22*** | 1.00 |  |
| Teacher support | 0.17*** | 0.21*** | 0.18*** | 1.00 |

****p*<0.0001
