# Supplementary material for: The role of social support in reducing the impact of violence on adolescents’ mental health in São Paulo, Brazil
Source: PLoS One. 2021 Oct 6;16(10):e0258036. doi: 10.1371/journal.pone.0258036 (PMC8494303; doi:10.1371/journal.pone.0258036)
Supplement: S2 Table — (DOCX) [file pone.0258036.s002.docx]

*S2 Table. Discriminant Validity of Social Support Scales (weighted)*

|  | Positive parenting | Parent involvement | Friend support | Teacher support |
| --- | --- | --- | --- | --- |
| q303 | **0.77** | 0.54 | 0.19 | 0.18 |
| q307 | **0.73** | 0.41 | 0.08 | 0.07 |
| q322 | **0.81** | 0.57 | 0.21 | 0.16 |
| q305 | 0.47 | **0.72** | 0.17 | 0.17 |
| q308 | 0.55 | **0.81** | 0.18 | 0.15 |
| q313 | 0.51 | **0.71** | 0.15 | 0.15 |
| q315 | 0.44 | **0.76** | 0.15 | 0.14 |
| q402 | 0.16 | 0.18 | **0.82** | 0.15 |
| q404 | 0.15 | 0.16 | **0.85** | 0.15 |
| q406 | 0.19 | 0.17 | **0.77** | 0.13 |
| q1802 | 0.13 | 0.14 | 0.16 | **0.80** |
| q1805 | 0.15 | 0.17 | 0.12 | **0.83** |
| q1808 | 0.13 | 0.17 | 0.15 | **0.78** |
